# Japanese Flounder pol-miR-155 Is Involved in *Edwardsiella tarda* Infection via ATG3

**DOI:** 10.3390/genes14050958

**Published:** 2023-04-22

**Authors:** Zhanwei Zhang, Xiaolu Guan

**Affiliations:** 1 CAS Key Laboratory of Experimental Marine Biology, Institute of Oceanology, Center for Ocean Mega-Science, Chinese Academy of Sciences, Qingdao 266071, China; 2 College of Marine Science and Engineering, Qingdao Agricultural University, Qingdao 266109, China

**Keywords:** MicroRNA, *Paralichthys olivaceus*, bacterial infection, autophagy, immune regulation

## Abstract

MicroRNAs (miRNAs) are small RNA molecules that function in the post-transcriptionally regulation of the expression of diverse genes, including those involved in immune defense. *Edwardsiella tarda* can infect a broad range of hosts and cause severe disease in aquatic species, including Japanese flounder (*Paralichthys olivaceus*). In this study, we examined the regulation mechanism of a flounder miRNA, pol-miR-155, during the infection of *E. tarda*. Pol-miR-155 was identified to target flounder ATG3. Overexpression of pol-miR-155 or knockdown of ATG3 expression suppressed autophagy and promoted the intracellular replication of *E. tarda* in flounder cells. Overexpression of pol-miR-155 activated the NF-κB signaling pathway and further promoted the expression of downstream immune related genes of interleukin (IL)-6 and IL-8. These results unraveled the regulatory effect of pol-miR-155 in autophagy and in *E. tarda* infection.

## 1. Introduction

MicroRNAs (miRNAs) are short regulatory RNAs that function in gene expression controlling at the post-transcriptional level [1,2]. Functional miRNAs were first discovered in the 1990s and attracted extensive attention from researchers since then. So far, large numbers of miRNAs and their functions have been examined [3,4,5]. MiRNAs function by completely or incompletely paring with the 3′-untranslated region (3′UTR) of target genes and participate in diverse biological processes including growth, apoptosis, and inflammation [6,7,8,9,10]. By manipulating diverse immune defending process, miRNAs were discovered to play differential roles in pathogen infection. MiRNA-30e-5p, a human miRNA induced by *Listeria monocytogenes (L. mono.)* infection, could target and depress the expression of suppressor of cytokine signaling (SOCS)1 and SOCS3, resulting in enhanced host immune response against infected bacteria [11]. In comparison, *Mycobacterium tuberculosis* induced miR-20a-3p in mice and depressed the release of proinflammatory factors by targeting I kappa B kinase β, which finally promoted bacteria survival [12]. MiRNAs related to immune responses against bacteria and virus infection have been reported in several fish species including ayu (*Plecoglossus altivelis*), grouper (*Epinephelus coioides*) and Japanese flounder (*P. olivaceus*) [13,14,15,16]. For example, miR-144, an up-regulated grouper miRNA in response to Singapore grouper iridovirus infection, could negatively regulate host immune response by targeting JNK3 and p38α mitogen-activated protein kinase (MAPK) and finally augment viral infection [17].

Autophagy is a conservative biological process that functions in maintaining homeostasis under stress such as energy loss or nutrient deprivation [18,19]. Autophagy serves as a degradation and recycling mechanism toward cargos including superfluous proteins and impaired organelles through the lysosome pathway [20,21]. Additionally, autophagy also acts as a vital cell-autonomous immune mechanism of the host to fight against bacterial and viral pathogens [22,23,24]. During autophagy, the invaded pathogens are captured by autophagic vesicles and transported to lysosomes for degradation. Some pathogens evolved to adopt diverse strategies to manipulate or hijack autophagy to benefit their replication [25,26]. The normal function of autophagy relies on the well-controlled autophagic flux that is regulated by various mechanisms, one of which is mediated by miRNA. In recent years, miRNA-dependent regulating strategies have been identified to play vital roles in autophagy [27,28,29]. For example, infection of *M. tuberculosis* induced the expression of the human miRNA, miR-889, that suppressed autophagy process via regulation of tumor necrosis factor (TNF)-like weak inducer of apoptosis (TWEAK), which resulted in a latent infection of *M. tuberculosis* [30]. In fish, miRNAs participating in autophagy regulation have been observed in several studies [31,32].

Autophagy-related genes (ATGs) are a group of executors and regulators of autophagy [33,34]. The well-controlled expression and function of ATGs are essential for normal autophagic flux. The first ATG was discovered in yeast in the 1990s [35]. Since then, large numbers of ATGs have been discovered, which function in various stages of autophagy, including nucleation of phagophore, elongation of double layer structure, formation of autophagosome and fusion with lysosome [36,37,38]. ATG3, with E2 enzyme activity, mainly functions in the elongation stage by promoting microtubule-associated protein light chain 3 (LC3) conjugation [39,40]. Deficiency of ATG3 resulted in defective autophagosome formation [41]. Depending on the autophagy system, ATG3 can participate in diverse processes, such as tumor progression, pathogens clearance and organelle homeostasis [42,43,44]. Previous studies proved that the infection of hemagglutinating virus of Japan envelope (HVJ-E) induced autophagy-dependent apoptosis in Hela cells, while knockdown of ATG3 with siRNA inhibited this autophagy-mediated apoptosis [45]. In addition, recent studies revealed several autophagy-independent functions of ATG3, e.g., involvement in DNA damage-induced mitosis [46].

*E. tarda* is an important aquatic pathogen for many species, in particular cultured fish such as Japanese flounder [47]. The *E. tarda*-associated disease led to severe mortality to flounder, causing great economic losses to the flounder industry. Understanding the mechanism of *E. tarda* pathogenicity will provide a theoretical basis for effective prevention of the *E. tarda*-associated disease. Previous reports showed that *E. tarda* infection of flounder induced the expression of diverse flounder miRNAs, some of which are predicted or proven to participate in autophagy [48]. Since autophagy is a multi-step process involving a large number of genes, we wondered how many of these genes might be indeed regulated by *E. tarda*-induced miRNAs. In this study, we examined flounder pol-miR-155, which was identified as an *E. tarda*-induced miRNA and predicted to target ATG3 [49], the regulator of autophagosome formation. Our study indicated that pol-miR-155 was a modulator of autophagy via ATG3 and also had a significant influence on *E. tarda* infection. The results of this study add new insights into the complex mechanism of *E. tarda*-induced regulation of fish autophagy via miRNAs.

## 2. Materials and Methods

### 2.1. Animals

Japanese flounders were purchased from a fish farm in Shandong Province. Before formal experiments, the fish were raised in seawater supplemented with oxygen at 22 °C for two weeks and confirmed as pathogen-free. Before tissue collection, the fish were euthanized with 0.1 g/L tricaine methane sulfonate as previously reported [50].

### 2.2. Cell Lines

The gill epithelial cell line FG-9307 [51] of Japanese flounder was cultured in Leibovitz’s L-15 medium (Sigma, St. Louis, MO, USA) plus 10% fetal bovine serum (FBS), 100 units/mL penicillin and 100 μg/mL streptomycin at 24 °C. HEK293T cells (CCTCC, Wuhan, China) were cultured in DMEM (Hyclone, Logan, UT, USA) with the same concentration of FBS and antibiotic as above under 37 °C and 5% CO_2_ conditions.

### 2.3. In Vivo Infection of E. tarda

*E. tarda* was cultured to an OD_600_ of 0.7 in LB broth under 28 °C as previously reported [52]. The bacteria were centrifuged at 8000 rpm for 3 min and washed with aseptic PBS three times. The bacteria were then suspended with aseptic PBS and diluted to 5 × 10^6^ CFU/mL. Before infection, fish were randomly separated into two groups. The fish in the infection group were intramuscularly injected with 100 μL bacteria suspension, while the fish in the un-infected group (Control) were injected with 100 μL PBS. At 24 and 48 h post-infection, the head kidney and spleen of experimental fish were collected and used for total RNA extraction.

### 2.4. Intracellular Replication Assay of E. tarda

*E. tarda* was grown to an OD_600_ of 0.7 as above. Before infection, FG-9307 cells were passaged into 24-well plates and cultured overnight. Prior to infection, the medium of FG-9307 cells was removed, and the cells were washed with aseptic PBS three times to clean up the residual antibiotics. The adherent cells were maintained in a fresh L-15 medium and then incubated with *E. tarda* of 5 MOI for 1 h and then incubated with 200 ng/μL gentamicin for 1 h after being washed with aseptic PBS. After washing with PBS, the cells were finally maintained in a L-15 medium plus 20 ng/μL gentamicin. At 2, 4 and 6 h post-infection, the cells were harvested to examine intracellular bacterial number by plate counting.

### 2.5. Quantitative Real-Time PCR (qRT-PCR)

To examine the influence of *E. tarda* on gene expression, FG-9307 cells were infected with bacteria as above. At 2 and 4 h post-infection, the cells were collected for total RNA extraction with RNA-easy^TM^ Isolation Reagent (Vazyme, Nanjing, China). The cDNA of pol-miR-155 were specifically reverse-transcribed with a stem-loop reverse transcription method using primer pol-miR-155-RT (Table 1). The cDNA was synthesized using First Strand cDNA Synthesis Kit (ToYoBo, Osaka, Japan). qRT-PCR analysis of pol-miR-155 and ATG3 expression was performed using comparative threshold cycle method (2^−ΔΔCT^) as previously reported [16]. The PCR procedure was as follows: 1 cycle at 95 °C for 30 s; 40 cycles of 95 °C for 10 s and 60 °C for 30 s. The primers used here are listed in Table 1. To determine the influence of pol-miR-155 on the expression of ATG3 and immune related genes, qRT-PCR analysis was performed in pol-miR-155 overexpressed FG-9307 cells. Briefly, pol-miR-155 mimic was transfected into FG-9307 cells using lipofectamine™ 3000 transfection reagent (Invitrogen, Carlsbad, CA, USA). At 24 h post-transfection, total RNA of the cells was extracted, and qRT-PCR analysis was performed as above to determine the influence of pol-miR-155 on ATG3, IL-6 and IL-8 expression. To evaluate the influence of in vivo *E. tarda* infection on gene expression, cDNA was transcribed from the total RNA of *E. tarda* infected flounder kidney and spleen. Expression of ATG3, IL-6 and IL-8 were determined as above.

### 2.6. Luciferase Reporter Assay

For determining the interaction between pol-miR-155 and ATG3, the luciferase reporter plasmid (named pATG3-Report) containing the 3′UTR of ATG3 was constructed. Briefly, the 3′UTR of ATG3 was amplified by PCR with the primer pair 3UTR-ATG3-F/3UTR-ATG3-R (Table 1). The PCR product was purified with E.Z.N.A.^®^ Gel Extraction Kit (Omega-bio-tek, Norcross, GA, USA) according to the manufacturer’s instruction. The obtained 3′UTR of ATG3 was inserted into the luciferase reporter plasmid of pmirGLO (Promega, Madison, WI, USA) with ClonExpress^®^ II One Step Cloning Kit (Vazyme, Nanjing, China). For the luciferase reporter assay, HEK293T cells were passaged into 24-well plates and transfected with pATG3-Report alone (Control), pATG3-Report plus pol-miR-155 mimic, pATG3-Report plus miR-NC or pATG3-Report plus pol-miR-155-M (pol-miR-155 with mutated seed sequence) with lipofectamine™ 3000 transfection reagent (Invitrogen, Carlsbad, CA, USA). At 24 h post-transfection, the transfected HEK293T cells were harvested, and the luciferase activity of different groups was determined using the Dual Luciferase Reporter Assay Kit (Vazyme, Nanjing, China). To analyze the influence of pol-miR-155 on NF-κB pathway activity, FG-9307 cells were transfected as above with the NF-κB reporter plasmid of pNF-kB-TA-Luc (Miaoling, Ji’nan, China) and the internal reference plasmid of TK plus pol-miR-155, miR-NC or DEPC-treated water (Control). At 36 h post-transfection, the luciferase activity of different group was detected as above.

### 2.7. Western Blot

To verify the regulation of pol-miR-155 on ATG3 expression, the western blot assay detecting the protein level of ATG3 was performed. FG-9307 cells were transfected as above with or without (Control) pol-miR-155 mimic or miR-NC. At 24 h post-transfection, the cells were harvested, and the protein level of ATG3 was determined by western blot as previously reported [48]. Briefly, the cells were lysed with RIPA buffer (Beyotime Biotechnology, Beijing, China) on ice for 30 min and centrifuged at 12,000 rpm for 5 min. The supernatants of cell lysates were mixed with 5 × loading buffer and boiled for 10 min. The samples were separated by SDS-PAGE, and the proteins were transferred to a nitrocellulose filter (NC) membrane. The NC membrane loaded with proteins was blocked with skim milk and incubated first with anti-ATG3 antibody (Abclonal, Wuhan, China) and then with Horseradish Peroxidase (HRP) conjugated secondary antibody (ABclonal, Wuhan, China). The HRP-linked protein bands were detected using ECL substrate (Beyotime Biotechnology, Beijing, China) and scanned with GelDoc XR System (Bio-Rad, Irvine, CA, USA). For examining the effect of pol-miR-155 and ATG3 on autophagy, FG-9307 cells were transfected with or without (Control) pol-miR-155 mimic, miR-NC, siRNA-ATG3 or siRNA-NC as described above. At 24 h post-transfection, autophagy activity of different group was determined by detecting LC3-Ⅱ formation via western blot as previously reported [52]. The β-actin (Abclonal, Wuhan, China) and α-tubulin (Abclonal, Wuhan, China) were used as internal references.

### 2.8. Effect of pol-miR-155 and ATG3 on E. tarda Infection

To examine whether *E. tarda* infection was influenced by pol-miR-155, the intracellular replication ability of *E. tarda* in pol-miR-155-overexpressed FG-9307 cells was determined. Briefly, FG-9307 cells in a 24-well plate were transfected with or without (Control) pol-miR-155 mimic or miR-NC. At 24 h post-transfection, *E. tarda* infection was performed as described above, and the bacterial number within cells was detected by plate counting. To detect the effect of ATG3 on *E. tarda* infection, FG-9307 cells were transfected with or without (Control) siRNA-ATG3 or siRNA-NC. At 24 h post-transfection, the intracellular replication ability of *E. tarda* was similarly determined as above.

### 2.9. MiRNA Mimic and siRNA

Mimic of pol-miR-155, pol-miR-155-M (with the seed sequence of 5′-UAAUGCU-3′ mutated to 5′-AUUACGA-3′) and miR-NC (the negative control miRNA) were synthesized by GenePharma (Shanghai, China). SiRNA-ATG3 (a siRNA targeting ATG3) was designed and synthesized by GenePharma. The negative control siRNA, siRNA-NC, was synthesized by the same company. All the synthesized miRNAs and siRNAs were dissolved in DEPC-treated water.

### 2.10. Statistical Analysis

All experiments were repeated at least three times. Data analysis was conducted with Student’s *t*-test using GraphPad Prism 5 (GraphPad Software, San Diego, CA, USA), and statistical significance was defined as *p* values < 0.05.

## 3. Results

### 3.1. The Expression of pol-miR-155 and ATG3 during E. tarda Infection

**Figure 1:**
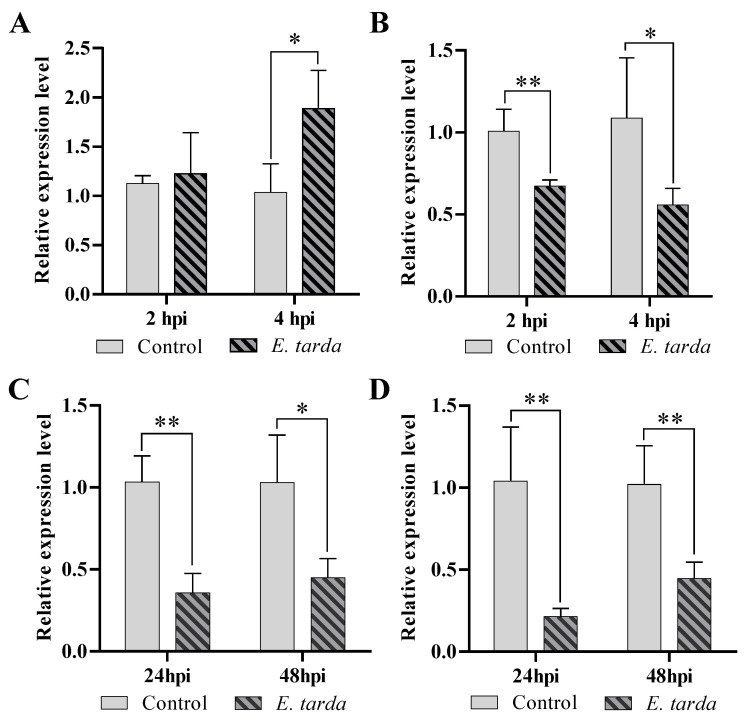
Regulation of pol-miR-155 and ATG3 expression by *E. tarda* infection. (**A**,**B**) FG-9307 cells were challenged with *E. tarda* for 2 h and 4 h, and the mRNA levels of pol-miR-155 (**A**) and ATG3 (**B**) were evaluated by qRT-PCR. (**C**,**D**) In vivo infection of *E. tarda* was conducted in Japanese flounder, and ATG3 expression levels in kidney (**C**) and spleen (**D**) were detected at 24 hpi and 48 hpi. Values are the means of four replicates and shown as means ± SD. ** *p* < 0.01, * *p* < 0.05.

Pol-miR-155 is a flounder miRNA regulated by megalocytivirus infection and predicted to target ATG3 by bioinformatic analysis [48]. To determine whether the expression of pol-miR-155 and ATG3 responded to *E. tarda* infection, the expression pattern of pol-miR-155 and ATG3 were examined in FG-9307 cells. Results showed that the expression of pol-miR-155 increased significantly at 4 hpi (Figure 1A), while in contrast, the expression of ATG3 decreased significantly at 2 and 4 hpi (Figure 1B). In flounder infected by *E. tarda*, the expression of ATG3 in kidney (Figure 1C) and spleen (Figure 1D) markedly decreased at 24 and 48 hpi.

### 3.2. Effects of pol-miR-155 and ATG3 on E. tarda Infection

**Figure 2:**
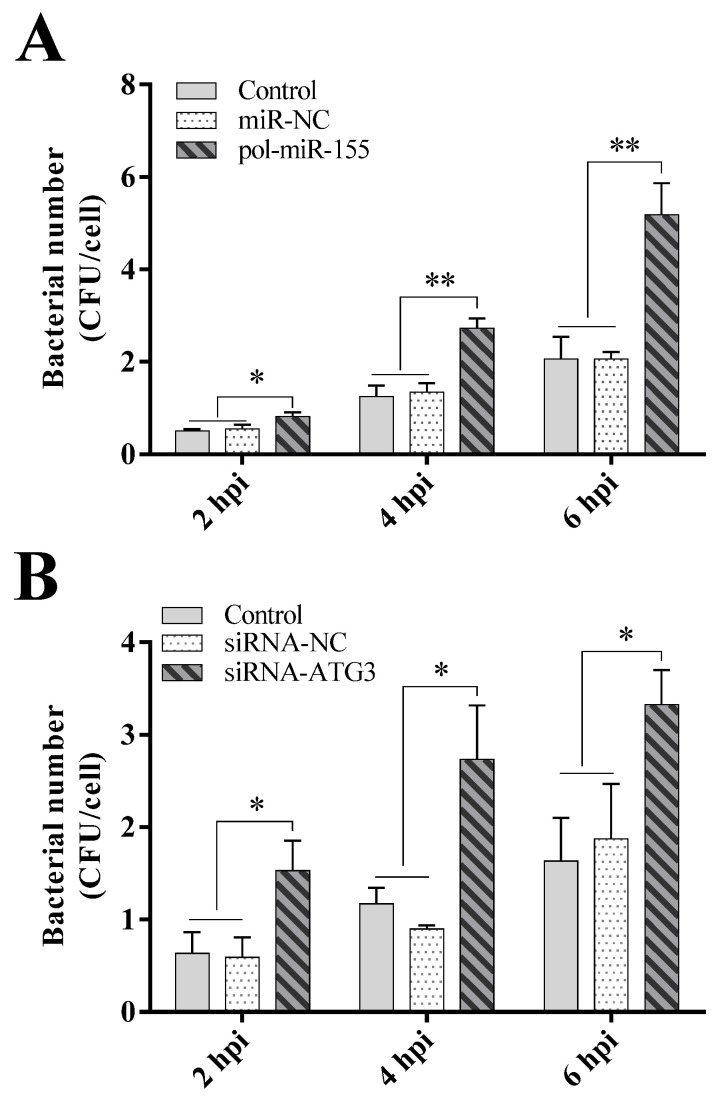
Effects of pol-miR-155 and ATG3 on *E. tarda* infection. (**A**) FG-9307 cells were transfected with or without (Control) pol-miR-155 or miR-NC for 24 h, followed by *E. tarda* infection. Intracellular bacterial load was detected at 2, 4 and 6 h post-infection (hpi) and shown as CFU (colony forming unit). (**B**) FG-9307 cells were transfected with or without (Control) siRNA-ATG3 or siRNA-NC for 24 h, followed by *E. tarda* infection. Intracellular bacterial load was detected as above. In all panels, values are the means of three replicates and shown as means ± SD. ** *p* < 0.01, * *p* < 0.05.

Having determined the expression response of pol-miR-155 and ATG3 to *E. tarda* infection, we further evaluated the effect of pol-miR-155 and ATG3 on *E. tarda* infection. Results showed that, in FG-9307 cells transfected with pol-miR-155, the intracellular bacterial number significantly increased at 2, 4 and 6 hpi, in comparison with the control cells or cells transfected with the negative control miRNA, miR-NC (Figure 2A). Consistently, FG-9307 cells transfected with the ATG-targeting siRNA (siRNA-ATG3) showed significantly higher intracellular bacterial growth at 2, 4 and 6 hpi than control cells or cells transfected with the negative control siRNA, siRNA-NC (Figure 2B).

### 3.3. Identification of ATG3 as a Target Gene of pol-miR-155

**Figure 3:**
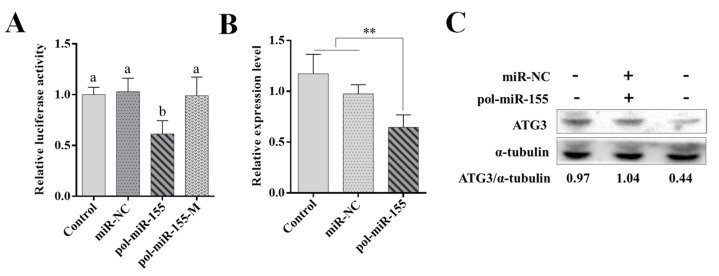
Regulation of ATG3 by pol-miR-155. (**A**) HEK293T cells were transfected with pATG3-Report, along with or without (Control) pol-miR-155 or miR-NC. Relative luciferase activities were detected at 24 h post-transfection (hpi). (**B**,**C**) FG-9307 cells were transfected with or without (Control) pol-miR-155 or miR-NC, and mRNA (**B**) and protein (**C**) levels of ATG3 were evaluated with qRT-PCR and western blot, respectively, at 24 hpi. Values are the means of four replicates and shown as means ± SD. ** *p* < 0.01. Different letters indicate statistical significance (*p* < 0.05).

Pol-miR-155 was predicted to target flounder ATG3. We therefore determined the interaction between pol-miR-155 and ATG3 3′UTR by luciferase reporter assay. Results showed that the luciferase activity in the pol-miR-155 overexpressing cells significantly decreased, with a reduction by 39%, in comparison with that in the control cells (Figure 3A). The luciferase activity in the negative control cells or the cells overexpressing pol-miR-155-M (a mutant pol-miR-155 with altered seed sequence) showed no significant change (Figure 3A). To further analyze the effect of pol-miR-155 on ATG3, pol-miR-155 was transfected into FG-9307 cells, and the endogenous expression of ATG3 was examined. Results showed that in comparison with the control group, the mRNA level of ATG3 in pol-miR-155 transfected group significantly decreased (Figure 3B). Consistently, the protein level of ATG3 in the pol-miR-155 transfected group was also reduced (Figure 3C).

### 3.4. Effects of ATG3 and pol-miR-155 on Autophagy

**Figure 4:**
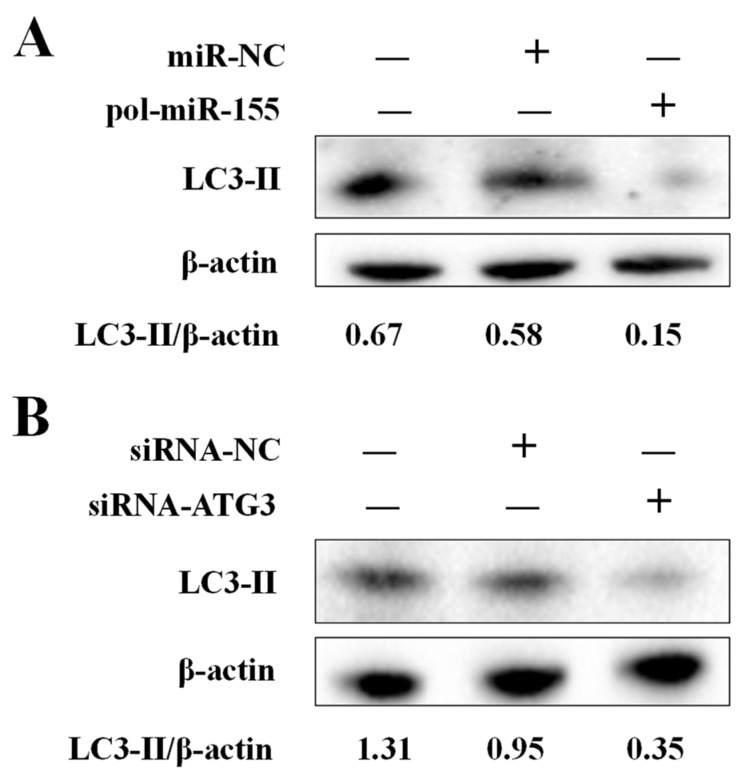
The effect of pol-miR-155 and ATG3 on autophagy. (**A**) FG-9307 cells were transfected with or without (Control) pol-miR-155 or miR-NC for 24 h, and LC3-II protein was determined using western blot. (**B**) FG-9307 cells were transfected with or without (Control) siRNA-ATG3 or siRNA-NC for 24 h, and LC3-II protein was determined by western blot. β-actin was used as a loading control. The relative densities of LC3-II/β-actin are shown at the bottom of the figures.

ATG3 plays a key role during autophagy in mammals, but the function of ATG3 in flounder autophagy is unknown. To evaluate the effect of ATG3 and pol-miR-155 on autophagy, knockdown of ATG3 or overexpression of pol-miR-155 was conducted in FG-9307 cells, and the autophagy marker protein of LC3 was detected by western blot. Results showed that, in pol-miR-155 overexpressed FG-9307 cells, the formation of LC3-Ⅱ, which reflects the activation of autophagy, was significantly depressed in comparison with the control group or negative control group (Figure 4A). Similarly, in ATG3 knockdown cells, the formation of LC3-Ⅱ was also inhibited (Figure 4B).

### 3.5. The Influence of E. tarda and pol-miR-155 on the Activity of the NF-κB Pathway

**Figure 5:**
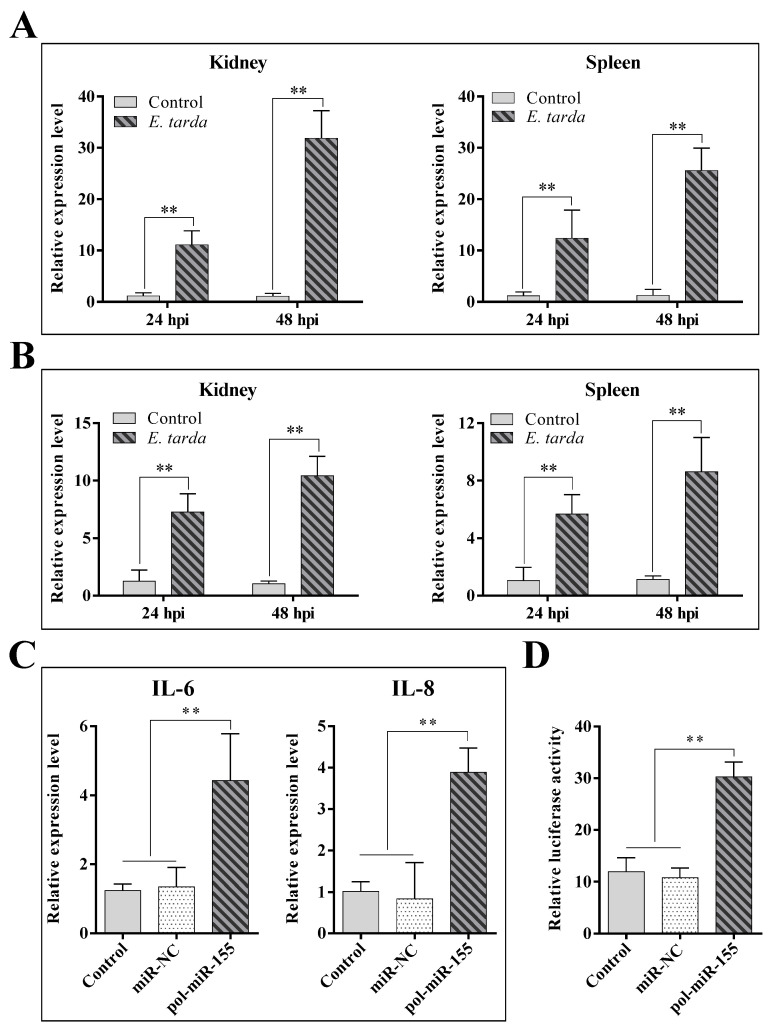
Influence of *E. tarda* and pol-miR-155 on immune-related gene expression and NF-κB activity. (**A**,**B**) Japanese flounders were infected by *E. tarda* for 24 or 48 h, and the expression of IL-6 (**A**) and IL-8 (**B**) in kidney and spleen was determined by qRT-PCR. (**C**) FG-9307 cells were transfected with or without (Control) pol-miR-155 or miR-NC for 24 h, and the expression of IL-6 and IL-8 was determined as above. (**D**) FG-9307 cells were co-transfected with pNF-kB-TA-Luc and TK plasmid, along with or without (Control) pol-miR-155 or miR-NC. Relative luciferase activities were determined at 36 h post-transfection. In all panels, values are the means of four replicates and shown as means ± SD. ** *p* < 0.01.

qRT-PCR analysis showed that in vivo infection of *E. tarda* significantly upregulated the expression of IL-6 (Figure 5A) and IL-8 (Figure 5B) in flounder kidney and spleen. Since *E. tarda* also induced the expression of pol-miR-155, we determined the effect of pol-miR-155 on the expression of IL-6 and IL-8. The results showed that the expressions of IL-6 and IL-8 in pol-miR-155 overexpressed cells significantly increased by 3.6-fold and 3.8-fold, respectively, compared with the control group (Figure 5C). As IL-6 and IL-8 are both downstream factors of NF-κB signaling, we examined the effect of pol-miR-155 on the activation of the NF-κB pathway using a luciferase reporter system. Results showed that overexpression of pol-miR-155 markedly enhanced the luciferase activity in FG-9307 cells, with a 2.6-fold increase in comparison with that in the control cells (Figure 5D).

## 4. Discussion

MiRNAs are crucial regulators of diverse biological processes including immunity. A large number of miRNAs have been identified to express differentially during pathogen invasion [53,54,55]. In fish, recent studies indicated that some miRNAs are responsive to both bacterial and viral pathogens [56]. Similarly, in this study, we found that the flounder miRNA of pol-miR-155, which was induced during megalocytivus infection [48], was also induced during *E. tarda* infection. MiRNAs generally regulate target gene expression by interacting with the 3′UTR of the target genes. In this study, ATG3 was predicted to be a target gene of pol-miR-155, and indeed, pol-miR-155 proved to interact with the 3′UTR of ATG3. Consistently, pol-miR-155 had a significant inhibitory effect on the mRNA expression and protein production of ATG3. Together these results confirmed ATG3 as the target gene of pol-miR-155.

During the battle between host and pathogen, miRNAs play intricate roles by exerting varied regulations on the immune response. MiRNAs can be employed by the host to clean up the invaded pathogen, thus maintaining host homeostasis. Meanwhile, pathogens can in turn hijack the miRNAs and use them as a weapon to attack the host immune defending system, thus promoting pathogen invasion [57]. For example, during *Listeria monocytogenes* infection, miR-21 was upregulated in mice, and miR-21 then targeted MARCKS and RhoB, which depressed *L. monocytogenes* intracellular replication [58]. In contrast, *Salmonella enterica* promoted the expression of miR-128, which inhibited macrophage recruitment by targeting CSF, leading to enhanced bacterial invasion [59]. Similarly, miR-24 induced by *Brucella* could negatively regulate STING expression, resulting in augmented intracellular infection [60]. As an intracellular pathogen, *E. tarda* can adopt various strategies to break host immune defense [61,62]. In this study, we found that *E. tarda* upregulated the expression of pol-miR-155, which then promoted *E. tarda* replication in flounder cells. These results suggested that pol-miR-155 may serve as a manipulating target for *E. tarda* to facilitate infection.

Autophagy is a self-digestion process with multiple functions. It is also an anti-infection strategy. During infection, the invaded pathogen is engulfed by the autophagic structure and finally degraded via the lysosomal pathway [63]. The autophagy process initiates with formation of phagophore, followed by elongation of the double-layer membrane to form phagosome and finally the formation of autophagosome [20]. During the elongation process, LC3 acts as a key component of the double layer membrane and functions in autophagic substrate recruitment [64]. The formation of functional LC3-Ⅱ relies on several autophagy related genes, one of which is the E2-conjugating enzyme ATG3. ATG3 is recruited by ATG12 and interacts with the activated LC3-Ⅰ to facilitate the conjugation of phosphatidylethanolamine (PE) to LC3-Ⅰ. In flounder, no report on the association of ATG3 with autophagy has been documented. In the present study, we found that flounder ATG3 was a target gene of pol-miR-155, and that overexpression of pol-miR-155 or knockdown of ATG3 attenuated the autophagy activity. These results suggested a conserved role of fish ATG3 in autophagy. The pro-bacterial infection effect of pol-miR-155 is probably due to repressed autophagy as a result of inhibition of ATG3 expression.

In mammals, miR-155 is known to exhibit an intricate connection with the NF-κB signaling pathway [65,66]. It acts as an NF-κB responsive miRNA, and stimulated overexpression of miR-155 could be blocked by inhibitor of the NF-κB pathway [67]. When delivered through serum exosomes to mice macrophages, miR-155 promoted NF-κB activation and further induced the production of TNF-α and IL-6 [68]. In contrast, overexpression of miR-155 in mice GBM cells attenuated the NF-κB signaling pathway by targeting and negatively regulating AGTR1 expression [69]. In this study, we observed that flounder IL-6 and IL-8, two typical NF-κB downstream factors, were induced by *E. tarda* infection and pol-miR-155. Furthermore, pol-miR-155 was able to activate NF-κB. These results suggested that *E. tarda* regulated the expression of immune-related genes via pol-miR-155-mediated NF-κB activation.

## Figures and Tables

**Table 1:** Primers used in this study.

Primers	Sequence (5′-3′) ^a^
3UTR-ATG3-F	TCTAGTTGTTTAAACGAGCTCACACATAGAGATGAAACT
3UTR-ATG3-R	CCTGCAGGTCGACTCTAGAGTCACAGTCTGTACAGAC
pol-miR-155-RT	GTCGTATCCAGTGCAGGGTCCGAGGTATTCGCACTGGATACGACACCCCT
pol-miR-155-F	CGCGTTAATGCTAATCGTGAT
pol-miR-155-R	AGTGCAGGGTCCGAGGTATT
ATG3-qRT-F	AAACAGATGAGGCGACCCTG
ATG3-qRT-R	GAGTCGAGGGGTCTGGTAGT
IL-6-qRT-F	CTCCAGTCGAATACGAGCCC
IL-6-qRT-R	ACTCTTTCTGGTGGTGAGCG
IL-8-qRT-F	GCCTGAGAAGCCTAGGAGTG
IL-8-qRT-R	TGACTCTCTTCACCCACGGA

^a^ Underlined nucleotides are restriction sites.

## Data Availability

The data presented in this study are available in the article.
